# The Long-Term Impact of Childhood Disability on Mental Health in Mid- to Late-Adulthood

**DOI:** 10.1093/geroni/igab046.3127

**Published:** 2021-12-17

**Authors:** Jessica West

**Affiliations:** Duke University Aging Center, Durham, North Carolina, United States

## Abstract

For decades, life course and stress process scholars have documented that negative, stressful experiences in childhood have consequences for health across the life course. One aspect of the childhood adversity that deserves more research attention is childhood disability. Children with disabilities experience higher levels of psychological distress compared to their peers and having a disability can negatively impact traditional markers of the transition to adulthood (e.g., education, employment, family status). At present, there is limited evidence regarding the impact of childhood disability on mental health over multiple years of adulthood. This study applies random effects models to nationally representative data from five waves (2008-2016) of the Health and Retirement Study (n=15,380; n=590 with a childhood disability), to examine how experiencing disability before the age of 16 shapes depressive symptoms over multiple years of adulthood. Given known gender differences in mental health, the models are stratified by gender to examine how the association between childhood disability and adult mental health varies by gender. Preliminary results suggest that experiencing a childhood disability is associated with different patterns of depressive symptoms in adulthood. Men who experienced childhood disability report more depressive symptoms in adulthood, net of sociodemographic, adult health, and childhood disadvantage covariates. Women who experienced childhood disability report more depressive symptoms in adulthood, net of all covariates except for childhood depression. Next steps are to conduct age-based growth curve models using Stata’s mixed function to estimate whether childhood disability influences baseline and growth of depressive symptoms in adulthood.

